# The Role of Cyclomodulins and Some Microbial Metabolites in Bacterial Microecology and Macroorganism Carcinogenesis

**DOI:** 10.3390/ijms231911706

**Published:** 2022-10-03

**Authors:** Natalia N. Markelova, Elena F. Semenova, Olga N. Sineva, Vera S. Sadykova

**Affiliations:** 1 Gause Institute of New Antibiotics, ul. Bolshaya Pirogovskaya, 11, 119021 Moscow, Russia; 2 Institute of Biochemical Technology, Ecology and Pharmacy, V.I. Vernadsky Crimean Federal University, 295007 Simferopol, Russia

**Keywords:** cyclomodulins, genotoxins, bacterial metabolites, microecology, carcinogenesis

## Abstract

A number of bacteria that colonize the human body produce toxins and effectors that cause changes in the eukaryotic cell cycle—cyclomodulins and low-molecular-weight compounds such as butyrate, lactic acid, and secondary bile acids. Cyclomodulins and metabolites are necessary for bacteria as adaptation factors—which are influenced by direct selection—to the ecological niches of the host. In the process of establishing two-way communication with the macroorganism, these compounds cause limited damage to the host, despite their ability to disrupt key processes in eukaryotic cells, which can lead to pathological changes. Possible negative consequences of cyclomodulin and metabolite actions include their potential role in carcinogenesis, in particular, with the ability to cause DNA damage, increase genome instability, and interfere with cancer-associated regulatory pathways. In this review, we aim to examine cyclomodulins and bacterial metabolites as important factors in bacterial survival and interaction with the host organism to show their heterogeneous effect on oncogenesis depending on the surrounding microenvironment, pathological conditions, and host genetic background.

## 1. Introduction

Epidemiological studies often reveal strong links between bacterial pathogens and cancer incidence: between *Helicobacter pylori* and gastric cancer; between *Salmonella typhi* and gallbladder carcinoma; between *Salmonella enteritidis* and colon carcinoma. Therein, many bacterial factors have been identified that mediate the malignant transformation of host cells: toxins, cell surface components, and effector proteins. Frequently, these factors target eukaryotic cell signaling pathways; interference with these pathways leads to cancer development [1]. Thus, many bacterial pathogens affect the Wnt/β-catenin signaling pathway, resulting in the induction of the β-catenin release. β-catenin penetrates the nucleus and activates the Wnt pathway genes to control the transcription of genes involved in apoptosis, cell proliferation, and malignant transformation [2,3]. Bacterial surface molecules can perform as activators, for example, *Fusobacterium nucleatum* adhesin FadA, which can bind to E-cadherin, inducing the release of β-catenin [4]. The *S. enteritidis* AvrA effector protein stabilizes β-catenin, which leads to Wnt signaling, and inhibits JNK and NF-κβ signaling pathways involved in inflammation and apoptosis [1]. The CagA toxin of *H. pylori* forms a functional CagA-c-Met-CD44 complex by activating Wnt/β-catenin, which induces the accumulation of nuclear β-catenin by activating PI3K/Akt signaling, resulting in the inhibition of apoptosis. *Bacteroides fragilis* toxin (BFT) causes dissociation of β-catenin from E-cadherin; the complex of β-catenin with TCF4 leads to c-Myc expression and cell proliferation [3]. No less significant bacterial factors associated with the malignant transformation of eukaryotic cells are toxins and effectors that cause changes in the eukaryotic cell cycle, for which they received their name—cyclomodulins. The cell cycle is one of the most favorable targets for toxins and effectors because basic cell functions are disrupted, which creates favorable conditions for bacterial invasion and colonization of the host. Cell cycle arrest at the G2/M phase by some cyclomodulins leads to disruption of cell renewal, induces delayed apoptosis of host cells, or blocks it. This increases the bacterial replication time and promotes colonization of the host, but at the same time can lead the cells to malignant transformation. Cyclomodulins of many commensal bacteria induce cell cycle arrest at the G2/M phase, creating a favorable environment for themselves without causing significant harm to the host [5].

The ecology of bacteria occupying the niches of mucous membranes and skin is associated with the colonization of these surfaces by commensals and pathogens, which, in the course of a long evolution, have developed various survival strategies both in the microbial environment and in the macro-organism [6]. Cyclomodulins are directly related to one of the survival strategies of bacteria. This strategy is based on causing limited damage to host cells, which disrupts their normal functions and defense against the pathogenic potential of the bacteria themselves. [7,8]. These cyclomodulins include cytotoxic necrotizing factors (CNFs), which prevent cytokinesis and simultaneously stimulate the cell cycle and DNA replication; cycle inhibitory factors (CIFs) that block the cell cycle and slow down apoptosis by inhibiting the proteasomal degradation pathway; DNA-damaging genotoxins, including a low-molecular-weight substance colibactin and protein cytolethal distending toxin (CDT) [9,10,11,12]. By modulating cell differentiation, apoptosis, and proliferation, cyclomodulins slow down the renewal of the epithelium, contributing to the prolonged colonization of macroorganism biotopes by the bacteria producing them. At the same time, eukaryotic cells preserved after exposure to toxins acquire genomic instability, which can play a certain role in their malignant transformation [13,14].

Unlike bacteria that adapt to the host through the production of toxins affecting its cells, there is a different microbial survival strategy—inhibition of competing microorganisms with the help of microbial metabolites. Bacteria producers of a number of known compounds, such as butyrate, lactic acid and secondary bile acids, colonize various biotopes of the human body supporting different physiological processes. However, under conditions of disruption of the homeostasis of the macroorganism by external and internal factors, microbial metabolism can affect the occurrence and/or development of pathological conditions, including neoplasms [15]. Among the carcinogenic metabolites of bacteria, the most studied are hydrogen sulfide and secondary bile acids, as well as reactive oxygen intermediates capable of causing oxidative damage to DNA. While the role of secondary bile acids in carcinogenesis is known, the role of butyrate, lactate, and other derivatives of short-chain fatty acids is described mainly as protective against cancer development [3]. At the same time, studies emerge that prove their contribution to carcinogenesis.

Strategies have evolved as microorganisms have adapted to specific host niches, leading to selective advantages and efficient survival of the microbes that acquire them. Despite the fact that the implementation of these strategies causes minor damage to the host and is associated only with the possibility of the spread of microorganisms, the human microbiota is increasingly considered an inducer or modulator of pathological processes, including many forms of cancer [16]. Most of the human population is colonized by tumor-associated microorganisms, but their presence does not mean that disease will develop in the future. Large-scale epidemiological studies are required to prove the carcinogenic effect of a large number of microorganisms with oncogenic potential. In most studies, the role of microorganisms in carcinogenesis is assessed experimentally in vitro and in vivo and based on the analysis of the microbiome of patients with cancer compared with healthy people. The most studied in the context of association with oncological diseases is the intestinal microbiota. [17,18,19].

Given the accumulated data on potential carcinogenic bacteria and their metabolic products, it is assumed that the manifestation of the carcinogenic properties of microbial toxins and metabolites depends on a combination of factors, such as the host’s genetic predisposition to cancer, chronic inflammatory processes, and the influence of the bio-environment [20].

In this review, we sought to understand the role of cyclomodulins and bacterial metabolites as factors in the survival and adaptation of bacteria to the host organism. Having assessed the positive role of these factors for the producing bacteria themselves, we have investigated possible changes in the host organism, in which the impact of cyclomodulins and microbial metabolites leads to the development of cancer. Finally, we have outlined the mechanisms and causes of tumor development and/or progression when eukaryotic cells are exposed to these compounds in vitro and in vivo.

## 2. Cyclomodulins—CNFs

CNFs are bacterial exotoxins of a protein nature that affect signal transduction in a eukaryotic cell, modulating cytokinetic processes. CNFs activate GTP-binding proteins of the Rho family (Rho GTPases): Rho (A, B, C) Rac, Cdc42 in cells via deamidation of glutamine (Q61 or Q63) at the Rho active site; as a result, Rho proteins lose the ability to hydrolyze the Rho-bound GTP [21,22]. Activation of Rho GTPase proteins blocks key regulators of cellular processes in their active state, causing changes in the actin cytoskeleton, cell proliferation, production of reactive oxygen species (ROS), and release of anti-apoptotic and pro-inflammatory factors [23,24].

CNF toxins are secreted and bind to eukaryotic cell surface receptors, causing endocytosis and subsequent translocation of the catalytic subunit into the cytosol. CNFs were found in *Escherichia coli*, *Yersinia pseudotuberculosis*, *Shigella* species, and *Salmonella enterica*. CNF1 is the most studied toxin produced by some strains of *E. coli* and is more common among uropathogenic *E. coli* living in the intestine and penetrating into the urinary tract through the urethra [9,22,25]. 

CNF toxins activate Rho GTPases, which, through the Akt/IkB kinase pathway, positively regulate the activity of NFkB, which in turn translocates from the cytoplasm to the nucleus and promotes transcription of the anti-apoptotic factors Bcl-2 and Bcl-XL and cell survival [7,26]. Protecting epithelial cells from apoptosis, CNF1 slows down their renewal, which leads to the colonization of the epithelium by toxigenic bacteria. Other important events that occur under the influence of toxin-activated Rho GTPases are changes in the actin cytoskeleton through actin polymerization and blocking of cytokinesis with the formation of multinucleated cells as a result of ongoing DNA replication. Infected cells develop lamellipodia and filopodia, endowing them with phagocytic behavior and the ability to macropinocytosis, facilitating the internalization of bacteria into host cells [23,27].

**Figure 1:**
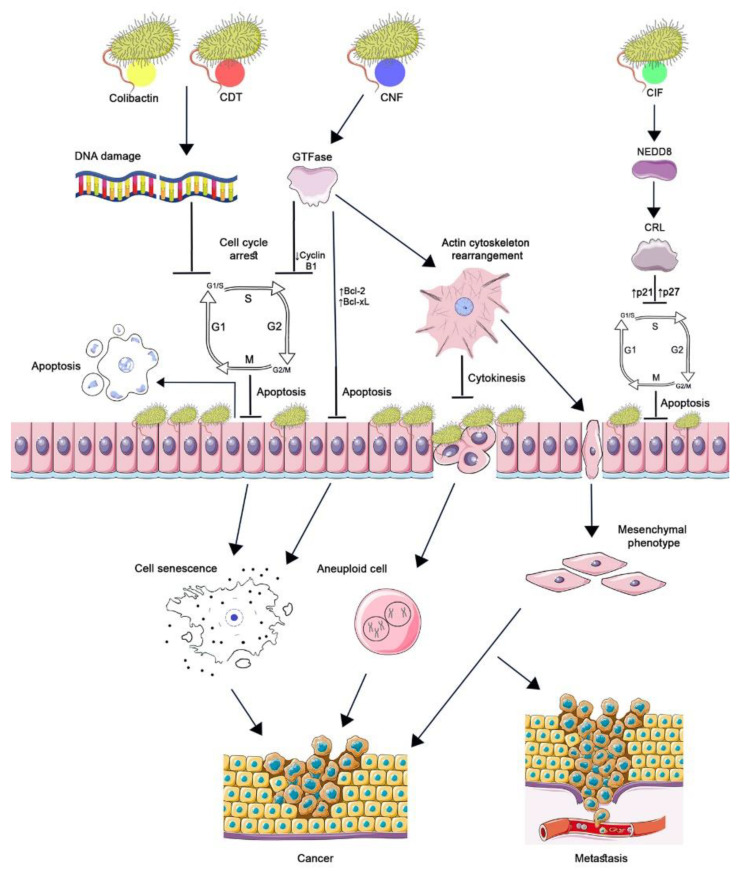
The role of cyclomodulins in carcinogenesis. CNF1, activating Rho GTPases, causes changes in the actin cytoskeleton, which leads to blocking of cytokinesis and the occurrence of aneuploidy and the epithelial-mesenchymal transition of cells. GTPases suppress the expression of cyclin B1 by arresting the cell cycle in the G2/M phase and increasing the transcription of Bcl-2 and Bcl-XL factors without triggering apoptosis. Aneuploidy, senescence, and the acquisition of a mesenchymal phenotype by cells lead to the development and progression of cancer. CIF, by binding to the ubiquitin-like protein NEDD8, inhibits the activity of CRL ubiquitin ligase. As a result, p21 and p27 accumulate in the cell, which leads to cell cycle arrest, prevention of eukaryotic cell proliferation, and delayed apoptosis. The genotoxins colibactin and CDT cause DNA damage resulting in cell cycle arrest at the G1/S and G2/M checkpoints. Cell growth and cell renewal arrest facilitate bacterial colonization. The cellular response to significant DNA damage consists either in the development of apoptosis or in the formation of a cellular senescence phenotype. Incomplete DNA repair of surviving cells leads to genomic instability, initiation of tumor development, and/or increased tumor growth. (Figure 1 was created using the images from Servier Medical Art https://smart.servier.com/ 26 June 2022).

CNF1′s potential role in carcinogenesis is also determined by its effect on eukaryotic cells (Figure 1). The absence of cytokinesis results in aneuploidy, which has been demonstrated by the treatment of the HCT 116 cell line with CNF1 toxin, the cell cycle reversibly arrested, and cell depolyploidization occurred. Their further entry into the cell cycle generated a large number of aneuploid progeny, which became more resistant to CNF1 [11]. Intercellular junctions disrupted under the influence of CNF1 improved the motility and migration of tumor cells, which can enhance their invasion and metastasis [23].

An experimentally in vitro epithelial-mesenchymal transition of intestinal epithelial cells induced by CNF1 was established. The acquisition of a mesenchymal phenotype by cells is currently recognized as one of the decisive factors in cancer progression [28]. It was also evident that GTPases activated by the Rho toxin can suppress the expression of cyclin B1; as a result, tumor cells (HeLa) are blocked in the G2/M phase without triggering an apoptotic response [29]. 

Despite the supposed role of the toxin in the development and/or progression of tumors, there are also records of the antitumor effect of CNF1 on mouse and human glioma cells, which leads to the blocking of cytokinesis of proliferating tumor cells, their aging, and death [30]. A recombinant CTX-CNF1 toxin that can penetrate the blood–brain barrier, selectively recognizes and affects glioma cells, and was created based on CNF1 and chlorotoxin (CTX) obtained from the venom of the scorpion *Leiurus quinquestriatus* [31].

Despite the specific activity of CNF1 against Rho GTPases and their permanent activation as a result of the constitutive association of Rho GTPase with GTP, some of the Rho pathways are activated temporarily. In this case, Rho proteins are depleted as a result of their ubiquitin-mediated proteasome degradation, which depends on the cell type. Thus, in HUVEC endothelial cells, macrophages, keratinocytes, fibroblasts, and 804G epithelial cells, the level of Rho ubiquitination is high, while in HEp-2, Vero, and HEK293 cells, it is low [26].

## 3. Cyclomodulins—CIFs

CIFs belong to bacterial cyclomodulins with enzymatic activities. The injection of CIF into the eukaryotic cell is carried out by the type III secretion system (T3SS), which transports various effector proteins into the host cell. CIF contributes to changes in eukaryotic cellular pathways in favor of the pathogen by arresting the cell cycle in the G1/S and G2/M phases. Irreversible cytopathic effects of CIF were first described in enteropathogenic *E. coli* (EPEC) and enterohemorrhagic *E. coli* (EHEC) in an infection model on cultured HeLa cells [32]. 

CIF homologs were found in other bacteria of vertebrates (e.g., *Burkholderia pseudomallei* and *Y. pseudotuberculosis*) and invertebrates (e.g., *Photorhabdus luminescens* and *Photorhabdus asymbiotica)*, and it has been shown that CIFs are conserved cyclomodulin proteins due to the presence of a conserved catalytic triad (Cys109-His165-Gln185). When the residues of this triad mutate, the cytopathic effect of CIF is not manifested [32,33].

CIF is encoded by a lambda-like prophage, and the cif gene may have spread widely through horizontal gene transfer. It is assumed that prophages encoding CIF are positively selected in bacteria to give them certain advantages in realizing their pathogenic potential. Thus, CIF in EPEC and EHEC is closely related to the LEE (locus of enterocyte effacement) pathogenicity island. CIF uses the existing T3SS, encoded by the LEE pathogenicity island, to be injected into target cells, and the resulting cytopathic effects slow down the turnover of affected cells. This gives selective advantages to LEE-positive *E. coli* over competing bacteria, especially in short-term adaptation processes [34].

Cell cycle arrest in the G2/M phase is associated with sustained CDK1 phosphorylation induced by CIF. At the same time, CIF is not a genotoxin and does not damage DNA like other cyclomodulins such as CDT or colibactin; therefore, it induces G2/M transition arrest regardless of activation of the DNA damage checkpoint pathway. CIF also blocks the entry of eukaryotic cells into the S phase, stopping the cell cycle in the G1/S phase. As a result of CIF action, CDK inhibitor proteins accumulate in cells: p21 inhibits the CDK1/CyclinB complex and the G2/M transition; p27, which, along with p21, inhibits the CDK2/CyclinE and A complexes and the G1/S transition [12,35].

Further studies have shown that the accumulation of p21 and p27 in infected cells is associated with the inactivation of CRL (Cullin-Ring ubiquitin Ligase), in which p21 and p27 are substrates of, and are degraded by, the ubiquitin-proteasome system. CIF, having deamidase enzymatic activity against NEDD8, a ubiquitin-like protein, impairs the conjugation of NEDD8 to Cullin, which leads to CRL inactivation. Thus, CIF inhibits ubiquitin-dependent degradation pathways of p21 and p27 proteins that regulate the cell cycle (Figure 1) [35,36].

The end result of inhibition of the proteasomal degradation pathway is cell cycle arrest prior to host cell destruction and apoptosis. By preventing proliferation, CIF can inhibit intestinal epithelial cell turnover, enhancing bacterial colonization. Delayed apoptosis or inhibition of apoptosis, associated, for example, with the accumulation of p21 in cells, also contributes to the local persistence of microorganisms. In addition, in an in vitro experiment, the time of death of cells exposed to CIF differed in various cell lines, which depended on the genetic background of the cells: HeLa cells died 72 h after infection; intestinal epithelial cells (IEC-6) with positive p53 died 48 h after infection [12,33].

Although, in in vitro experiments, CIF inhibited the proliferation of cancer cells and eventually led to apoptosis, where new epidemiological data confirm the role of CIF in carcinogenesis. In a recent multicenter case-control study, an association of the cif gene with precancerous lesions—intestinal polyps or adenomas—was found that can occur in the early stages of carcinogenesis, and this association has been assessed as a statistically significant risk factor [37].

## 4. Cyclomodulins—Genotoxins

Among the bacterial toxins, there is a unique group of genotoxins whose molecular target is DNA. The action of these toxins implements one of the strategies for the survival and adaptation of microorganisms to the ecological niche, aimed at causing damage to the host cells. Currently, three bacterial toxins are classified as genotoxins: polyketide toxin (colibactin), cytolethal distending toxin, and a toxin produced by *S. typhi* [38,39].

All eukaryotic cells respond to DNA damage and repair it, preserving the integrity of the genome. DNA breaks not eliminated during repair lead to cytogenetic disorders, oncotransformation, or cell death. Genotoxins cause single-strand or double-strand DNA breaks in target cells and trigger a signaling pathway that prevents the transition between phases of the cell cycle, resulting in its arrest, cellular senescence, or apoptosis [20,38].

Inhibition of cell proliferation and renewal by genotoxins promotes local bacterial colonization as a result of increased bacterial adhesion, which can occur due to changes in the distribution of receptors on the surface of affected cells with impaired cell morphology. Along with the cells, the functional capacity of the tissues composed of them and the local lymphocytes exposed to toxins also change, which can lead to bacterial invasion and infection [40]. Chronic inflammatory diseases are associated with an increased risk of developing tumors. In the case of bacterial genotoxins, there is an assumption that damaged DNA is the main factor in the acquisition of a malignant phenotype by cells and the onset and/or progression of a tumor, especially in combination with host genetic factors [41]. Figure 1 shows the main events that lead eukaryotic cells to develop a malignant phenotype under the influence of genotoxins.

Colibactin, a nonribosomal polyketide peptide, is a secondary metabolite found in various species of bacteria of the *Enterobacteriaceae* family, including *E. coli*, *Citrobacter koseri*, *Klebsiella pneumoniae*, and *Enterobacter aerogenes*, carrying the genomic island of polyketide synthase (pks) on the bacterial chromosome. The origin of pks is not entirely clear, but it has been hypothesized that the clb gene cluster spreads by horizontal transfer (HGT) through ICE-like elements (integrative and conjugative elements, ICEs) and undergoes a stabilization (homing) process upon their chromosomal integration [9,42].

The pks island was originally described in the extraintestinal pathogenic *E. coli*, ExPEC (Nougayrede et al., 2006) [43]. It has been shown that the presence of the pks island among *E. coli* is characteristic of the B2 phylogenetic group, which includes most of the ExPEC strains characterized by high virulence, such as expression of the K1 capsule in *E. coli* causing sepsis and neonatal meningitis [43,44]. The pks gene cluster in *K. pneumoniae* is identical to *E. coli* and is also associated with *K. pneumoniae* hypervirulence [45]. It is noteworthy that the systemic infection of *E. coli* K1 and *K. pneumoniae* K1 necessarily includes the invasion of the intestinal mucosa and the stage of intestinal translocation, which is reduced due to impaired colonization of the intestine by these bacteria upon inactivation of individual genes of the pks island-clbA or clbP, in experimental infections of animals [46,47]. The B2 group is associated with the carriage of many pathogenicity factors: adhesins (P- and Type 1 Fimbriae), capsular antigens (K1 and K5), aerobactin, hemolysin, and some pathogenicity islands (PAIs) associated with the persistence of *E. coli* in the intestinal microbiota. These factors increase the adaptability of bacteria to the normal intestinal environment, and the occurrence of extraintestinal virulence is an accidental by-product of commensalism [48]. The pks island is also often detected in intestinal isolates of *E. coli* extracted from healthy people, including infants, and is even present in the probiotic strain *E. coli* Nissle 1917 [49]. Probably, the acquisition of genotoxicity promotes increased colonization of the intestine by pks + bacteria, acting as a factor in the survival of bacteria that create and expand their own niches in the complex microbial ecosystem of the intestine [50,51]. Successful competition for host biotopes of microorganisms is also associated with their antimicrobial activity and intermediate products formed during the synthesis of colibactin, inhibiting the growth of other bacteria—*Staphylococcus aureus*, *Bacillus subtilis*—and giving an advantage to bacteria carrying pks genes [50,52].

The pks genomic island of *E. coli* contains 19 genes (from clbA to clbS), 54 kb in size, encoding the synthesis of colibactin, while the structure of the compound remains unknown due to its high instability. The structure of 13 synthetic derivatives of colibactin was proposed using a synthesis strategy, and it has been shown that the formation of an electrophilic cyclopropane fragment, which can bind to DNA and alkylate adenine of both strands, causing DNA interstrand cross-links, ultimately occurs. This phenomenon has been identified both in HeLa cells and in mouse models [53,54].

The genotoxic activity of colibactin depends on contact with human or animal cells and is not observed when cell lines are treated with pks + *E. coli* bacterial culture supernatants or their lysates. This contact is facilitated by inflammation when the mucus layer on the surface of the colon is broken, and the contact of the intestine’s surface cells with live pks + *E. coli* becomes possible [55,56]. Mucus becomes more permeable when mucosal homeostasis is disturbed, including excessive consumption of mucus glycans by microorganisms as an alternative source of nutrients. As a result, bacteria penetrate into the inner layers of mucus, and severe damage can lead to erosion of the colonic mucosa [57]. In addition, many intestinal bacteria interact with mucosal carbohydrates, glycoproteins, and glycolipids to colonize the mucosal surface. Toxin-producing bacteria may have a double advantage in intestinal colonization due to the fact that toxins also attach to host glycan structures. It is likely that the disturbed mucus layer can not only affect the pathogenic and carcinogenic potential of bacteria, but also their ability to penetrate the inner mucus layer and interact with transmembrane mucins covering the apical surface of enterocytes [58].

Subsequent changes in cells, once bacteria are in close contact with them, are associated with DNA double-strand breaks (DSB) accompanied by phosphorylation of replication protein A (pRPA) and histone H2AX (pH2AX), which occur due to replication stress caused by DNA cross-links. The ATM-CHK2 DNA damage checkpoint pathway is activated, and the cell cycle stops at the G2/M stage, resulting in subsequent incomplete DNA repair [50]. By damaging DNA and blocking the cell cycle, the renewal of eukaryotic cells is slowed down, which contributes to the stability and long-term persistence of pks + bacteria in the complex microbial communities of the host intestine [56]. Further events in cells damaged by colibactin can be considered as its side effect, promoting carcinogenesis. Surviving eukaryotic cells are characterized by genomic instability, expressed in chromosomal aberrations, aneuploidy, and an increase in the frequency of gene mutations [9,14]. The role of pks + *E. coli* in mutagenesis was confirmed by a study conducted on human intestinal organelles derived from primary crypt stem cells. In organelles exposed to pks + *E. coli*, mutational signatures of SBS-pks were revealed with an increased level of single base substitutions (SBS) in ATA, ATT, and TTT, with the replacement of the middle T and small indel, ID-pks, with single T deletions at homopolymers. In addition, upstream of the SBS-pks sites and upstream of the poly-T site in ID-pks, adenine enrichment was found, which is characteristic of pks signatures and distinguishes them from those of other alkylating agents. Notably, based on the analysis of WGS data from a Dutch collection of solid cancer metastases, SBS-pks and ID-pks signatures were detected mainly in CRC [59].

A dose-dependent effect of pks + *E. coli* on eukaryotic cells has been shown as follows: low doses induced a transient response to DNA damage followed by resumption of cell division with signs of damaged DNA; high doses of toxigenic bacteria caused irreversible cell cycle arrest and apoptosis [13]. A large number of pks + bacteria causes massive DNA damage and phenotypic cellular senescence, in which the cytotoxic effect of colibactin is observed—megalocytosis, the absence of mitosis [47,54]. Senescent cells begin to secrete pro-inflammatory cytokines, chemokines, proteases, and growth factors into the environment, such as hepatocyte growth factor (HGF), which stimulate the proliferation of uninfected neighboring cells. Therefore, pks + bacteria accelerate the progression of neoplasms, as shown in mouse models of colon tumors [60,61].

Thus, the putative role of colibactin in carcinogenesis may be associated with both the initiation of tumor development and enhancement of tumor growth. In support of this, colibactin-positive bacteria are found with increased frequency in patients with inflammatory bowel disease (IBD), familial adenomatous polyposis, and colorectal cancer (CRC) and are associated with the etiology and pathogenesis of these diseases [62,63]. Accumulating epidemiological evidence indicates the existence of dietary risk factors for colorectal cancer that are metabolized by the gut microbiota and may influence its composition. A high intake of grains or a low intake of vegetables, especially from the cruciferous family, revealed a positive association between pks + *E. coli* and colorectal neoplasia [19].

Infections of other organs by pks + bacteria are also considered to be an increased risk factor for cancer. Thus, bladder cancer may be preceded by regular urinary tract infections (UTIs) caused by *E. coli* pks + [11]. It has been shown that in the urine of patients infected with isolates of uropathogenic *E. coli* (UPEC) pks +, the C14-Asn metabolite, which is formed during the synthesis of colibactin, is detected, and damage by colibactin to the DNA of urothelial cells Krt14 from which a significant part of cancer tissue originates, was experimentally confirmed on rodents. [64].

Cytolethal distending toxin is a genotoxin produced by many Gram-negative bacteria, such as *E. coli, Campylobacter* spp., *Aggregatibacter actinomycetemcomitans, Haemophilus ducreyi, Helicobacter* spp., *Shigella dysenteriae, Haemophilus* spp., *Providencia alcalifaciens* [10,65,66]. CDT-producing bacteria colonize mucus membranes and skin as commensals or as agents of localized or disseminated infections in mammals [14]. Initially, CDT was detected in pathogenic *E. coli* upon treatment of Vero, CHO, HeLa, and HEp-2 eukaryotic cells with bacterial culture supernatant, which caused cell elongation and nucleus enlargement 120 h after addition [67].

Morphological changes associated with CDT are a consequence of cell cycle disturbances in infected cells. In most bacteria, the gene cluster encoding CDT subunits is located on the chromosome; in some *E. coli* strains, it is located on the conjugative plasmid (pVir), which also encodes other virulence determinants [68]. CDT consists of three different protein subunits, A, B, and C, which are encoded by three genes, cdtA, cdtB, and cdtC, respectively. They were first cloned and sequenced from *E. coli* (O86:H34) (Scott and Kaper, 1994) [69]. The cdtA subunit interacts with receptors on the surface of target cells and delivers the toxin into the cytosol; the cdtC subunit interacts with intracellular transport systems and ensures the transfer of the cdtB toxin into the eukaryotic cell nucleus. Once the cdtB enzyme translocates to the nucleus, it induces DNA single-strand and DSBs and cell cycle arrest at the G1/S or G2/M boundary, depending on the cell type [20,41]. The accumulation of DSB leads to a cellular response to DNA damage involving the ATM (ataxia telangiectasia mutated) system, which activates Chk2, which in turn phosphorylates and inactivates CDC25 phosphatase, resulting in the phosphorylated cyclin B-CDK1 complex preventing mitotic entry. Cell cycle arrest at the G1/S stage by CDT mainly depends on p53: ATM phosphorylates p53 and activates p21, which inhibits cyclin E–CDK2, which in turn blocks entry into the S phase [65]. The arrest of epithelial and endothelial cell lines mainly occurs in the G2/M phase, and the arrest of fibroblast cells in both the G1/S and G2/M phases of the cell cycle, with G1/S arrest observed in primary fibroblast cell lines containing wild-type p53 [10,70]. The subsequent event of CdtB-induced activation of the cellular response may be apoptosis mediated through both p53-dependent and p53-independent pathways. Hematopoietic cells undergo rapid p53-mediated apoptosis; epithelial, endothelial clones, and all p53-deficient cells show resistance to apoptosis, which leads to cellular senescence [71].

Limited damage to host cells stimulates specific cellular responses. Influencing the activity of B- or T-cells, CDT prevents their maturation into effector cells and induces the aging of T-cells in vivo [72]. Microorganisms producing CDT may be the cause of irritable bowel syndrome with diarrhea (IBS-D). Anti-cdtB antibodies cross-react with an intracellular cytoskeletal protein, vinculin, which is a component of cell adhesion and plays a key role in the contractility of nerve cells in the gastrointestinal tract. [73]. Pathogens such as *Campylobacter jejuni* or *E. coli*, which trigger the subsequent development of IBS-D, first cause gastroenteritis. The further effect of the toxin on the proliferating epithelial cells in the intestinal crypts leads to the cell cycle arrest in the G2 phase. As a result, cell growth and renewal stop, which facilitates the colonization of the intestine by bacteria. Impaired maturation of epithelial cells of intestinal villi leads to epithelial erosion and loss of absorptive function [39]. Persistent colonization and associated inflammation, invasion, immunosuppression, and DNA damage may influence the onset and development of tumors.

The role of CDT in oncogenesis was confirmed by experiments carried out on GF (germ-free) Apc ^Min/+^ mouse models (He et al., 2019) [74]. It has been shown that genotoxin produced by *C. jejuni* isolate extracted from human, due to the toxic effect of CdtB, induced DNA damage, contributing to the development of colorectal cancer, while the number and size of tumors in mice decreased when they were infected with bacteria with a mutated CdtB subunit [74]. In the intestinal and liver cell lines, CdtB-dependent regulation of the V-maf musculoaponeurotic fibrosarcoma oncogene homolog B (MAFB) gene has been found [75].

In vitro and in vivo experiments have also demonstrated the possible role of the toxin in tumor progression. In response to infection with bacterial CDT, cytoplasmic invaginations formed by the nucleoplasmic reticulum (NR) were found in target cells. NR structures, being the active sites of mRNA translation, correlated with a high degree of ploidy and were involved in the survival of cells with damaged DNA. Polyploid cells continued to proliferate with NR resorption and returned to normal size, which may contribute not only to the continued growth of the tumor, but also to its resistance to radiation therapy that causes DNA damage [66].

In some cases, the effect of CDT on cancer cells led to their accelerated death associated with the implementation of the strategy of genotoxin-infected cells’ apoptotic death. It turned out that CDT effectively inhibits the growth of radioresistant prostate cancer associated with the loss of DAB2IP expression. DAB2IP is a protein that activates Ras-GTPase and regulates proliferation and apoptosis by inactivating PI3K-Akt. The toxin enhances the effect of IR as a result of prolonged cell cycle arrest at G2/M followed by apoptosis induction [76].

## 5. Specific Microbial Metabolites

**Figure 2:**
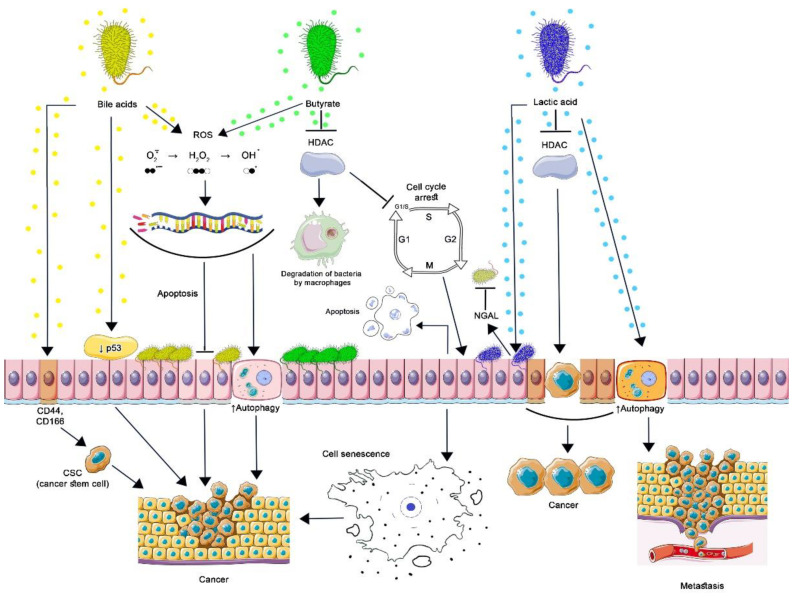
Contribution of commensal bacteria microbial metabolites to carcinogenesis. By stimulating the proteasomal degradation of p53 and inducing cancer stem cells (CSC), secondary BAs lead to malignant transformation of cells. Oxidative damage to DNA by secondary BA and butyrate is associated with the formation of ROS, which cannot be restored in genetically modified cells, which leads to the formation of resistance to apoptosis, increased autophagy, and cancer development. Butyrate inhibits HDAC in normal and cancer cells. The action of butyrate upon HDAC3 of intestinal macrophages reduces the activity of the mTOR protein, as a result of which autophagy in macrophages is enhanced. This is how butyrate-producing bacteria maintain the optimal level of their population. By inhibiting HDAC, butyrate induces the expression of p21, which arrests the cell cycle at the G1-S stage: cells can undergo apoptosis or acquire a senescence-associated secretory phenotype leading to the development or progression of cancer. By inhibiting the production of cyclic adenosine monophosphate (cAMP), lactic acid activates autophagy in vaginal epithelial cells, protecting them from intracellular microorganisms. Protective autophagy in tumor cells prevents apoptosis, promoting tumor metastasis. By blocking HDAC, lactic acid increases the production of NGAL by epithelial cells, which inhibits the growth of other microorganisms, inducing selective transcription of genes in malignantly transformed cells, which contributes to their survival and stimulates oncogenesis. (Figure 2 was created using images from Servier Medical Art https://smart.servier.com/ 26 June 2022).

Bacterial populations in the human body and specific metabolites associated with them support physiological functions in its various biotopes. Depending on the genetic and epigenetic background of the host, an imbalance between the protective and pathological functions of microbial metabolites can lead to the occurrence of diseases, including cancer [15,77]. Figure 2 shows the main events that lead eukaryotic cells to develop a malignant phenotype when exposed to bacterial metabolites.

Butyrate is an organic metabolite of the colon microbiota that plays a key role in maintaining homeostasis and regulating intestinal barrier function. The main producers of butyrate in the human intestine are obligate anaerobes belonging to phylogenetically diverse groups of bacteria: *Faecalibacterium prausnitzii* and *Roseburia* spp., which belong to *Ruminococcaceae* (*Clostridial* cluster IV) and *Lachnospiraceae* (*Clostridial* cluster XIVa) families of the phylum *Firmicutes*, respectively [78,79,80].

Energy metabolism and microbial ecology are common to groups of butyrate-producing bacteria; therefore, to maintain their populations, they compete with carbohydrate-consuming *Bacteroides* spp. and have an advantage at a lower pH (5.5) of the proximal colon. This moderately acidic pH level occurs when there is a high level of bacterial fermentation of carbohydrates in the colon, which are not digested in the upper intestine [81]. Butyrate has a direct antimicrobial effect on bacteria, damaging their cell membranes and acidifying the cytosol, as well as increasing the production of host defense peptides (HDP) by intestinal epithelial cells: RegIIIγ, β-defensins, cathelicidin LL-37 [82,83]. Acting as a differentiation factor, butyrate induces metabolic and transcriptional changes in monocyte-derived macrophages, enhancing their antimicrobial functions. These changes are associated with the action of butyrate as an inhibitor of histone deacetylases (HDAC)—enzymes that remove histone acetyl groups. HDAC inhibitors maintain a high level of histone acetylation at promoter loci, providing an epigenetic mechanism that regulates their expression. As a result of butyrate’s effect on HDAC3 of intestinal macrophages, they increase the expression of calprotectin, a protein that enhances the destruction of bacteria through zinc ions (Zn^2+^) and manganese (Mn^2+^) sequestration, decreases the activity of the mTOR protein, which stops the suppression of autophagy, and increases the production of LC3, a protein involved in the formation of autophagosomes [83,84]. Butyrate triggers a phagocytic antimicrobial defense program in the absence of an increased inflammatory cytokine response; in addition, butyrate reduces the production of pro-inflammatory mediators such as NO, IL-6, and IL-12. Thus, butyrate-producing bacteria, by regulating HDAC and avoiding the destructive effects of inflammation, contribute to maintaining the optimal level of their population in the intestine [84,85].

Butyrate plays an exceptional role in intestinal homeostasis and has an anticarcinogenic effect on cancer cells of various genesis, which is well described in numerous literature sources. But do the underlying mechanisms of the antitumor effect of butyrate lead to the expected result in all cases? We tried to figure that out.

Butyrate, as an HDAC inhibitor, has antiproliferative properties against tumor cells. Normal colonocytes use butyrate as the main source of energy, while cancerous colonocytes use glucose during glycolysis (Warburg effect). Therefore, butyrate can accumulate in the nucleus and function as an HDAC inhibitor, increasing the expression of target genes and preventing the proliferation of cancerous colonocytes [86]. For instance, inhibition of HDAC in tumor cell lines induces p21 expression. This inhibitor of cyclin E (cyclin-dependent kinase 2, Cdk2) activity stops the cell cycle at the G1-S stage, while non-proliferating cells differentiate or undergo apoptosis, which can be interpreted as effects that limit the occurrence and progression of cancer [87]. Although irreversible cell cycle arrest initially acts as a defense mechanism against carcinogenesis, it is believed that this leads to an accumulation of cells with a senescence-associated secretory phenotype (SASP), which can lead to tumor progression. CRC-associated butyrate producers *Porphyromonas gingivalis* and *Porphyromonas asaccharolytica* have been found to induce a senescence-associated secretory phenotype (SASP) in cultured intestinal epithelial cells and organelles as well as accelerate colorectal tumor development in mice. SASP cells contribute to the accumulation of immune cells in their environment and maintain chronic inflammation and disruption of the epithelial cell barrier integrity due to impaired tight junctions between them, which can contribute to carcinogenesis [88].

Differences in the host genotype can also lead to a multidirectional effect of butyrate on colonic epithelial cells, which has been demonstrated in experiments on mouse models. Butyrate stimulated the formation of colorectal polyps in mice with the APC ^Min/+^ genotype, MSH2 ^−/−^. Mice with multiple intestinal neoplasia, APC ^Min/+^) represent an animal model of human adenomatous polyposis. Furthermore, these mice were deficient in the DNA repair gene, 2 MutS homolog (MSH2 ^-/-^), which is involved in correcting errors that occur during DNA replication. Presumably, MSH2 deficiency intensifies genetic changes in the tumor, which can lead to increased cell proliferation in response to the action of butyrate [89,90].

Butyrate is actively produced by commensal bacteria and absorbed by colonocytes from the colonic lumen, then it undergoes β-oxidation until acetyl-CoA forms in mitochondria, maintaining energy homeostasis and preventing cell autophagy [78,79]. On the other hand, the mitochondrial oxidative metabolism of butyrate is the main mechanism that increases the amount of ROS in intestinal epithelial cells. During normal metabolism or inflammation, ROS-induced DNA damage in colonocytes is repaired by the BER (base excision repair) and MMR (mismatch repair) systems. However, in the absence of the MMR system in tissues, as shown in mouse models, butyrate leads to the accumulation of ROS and oxidative DNA damage. In the case of both Lynch syndrome and the sporadic form of CRC, MMR genes are defective and cannot repair damage caused by ROS, which leads to the accumulation of oxidative stress product, 8-oxo-dG, in the body [80].

Also interesting are the new data on the development of resistance to chemotherapy of CRC cells, which occurs from the resistance of CRC cells to butyrate. CRC patients responding to chemotherapy have a high abundance of butyrate-producing bacteria. Butyrate resistance is formed by the efflux mechanism as a result of an increase in the expression level of efflux genes. At the same time, this mechanism contributes to the formation of cross-resistance of HCT-116 cells to metformin and oxaliplatin and PMF-K014 cells to 5-fluorouracil [91].

Lactic acid. A significant part of the microbiota of various human loci is made up of bacteria that produce lactic acid, the main nutrient substrate for which is epithelial cell glycogen. Species *L. crispatus*, *L. iners*, *L. gasseri*, and *L. jensenii* produce especially large amounts of lactic acid in the vagina [92]. Lactobacilli produce lactic acid, which lowers the pH of the extracellular medium, creating favorable conditions for their own survival and preventing the adhesion and growth of other microorganisms [93]. The protonated form of lactic acid, which is permeable to membranes without the use of monocarboxylate transporters and the GPR81 receptor, has antimicrobial and immunomodulatory activity. Lactic acid, penetrating into bacterial cells, acidifies the cytosol and increases the permeability of the cell membrane, disrupting its intracellular functions, which leads to the death of bacteria [94].

The antimicrobial activity of lactic acid is aimed at potentially pathogenic microorganisms and different infectious agents, while lactic acid induces immune tolerance, reducing the activity of cytotoxic T lymphocytes and natural killer cells, inhibiting the release of pro-inflammatory cytokines from epithelial cells and the maturation of dendritic cells. Similarly, lactic acid, formed by tumor cells as a result of the predominance of anaerobic metabolism, creates a favorable tumor microenvironment by blocking the transcription of the interferon-γ gene, contributing to the weakening of antitumor immunity, inducing HIF-1α to enhance the proliferation of cancer cells, inducing the synthesis of hyaluronan by tumor cells to destroy extracellular matrix, and increasing their metastatic potential [95].

Lactic acid inhibits the production of cyclic adenosine monophosphate (cAMP), activating autophagy in vaginal epithelial cells, thereby protecting them from oxidative stress resulting from the intracellular accumulation of defective mitochondria, as well as from intracellular microorganisms [96,97]. Despite the fact that autophagy is a mechanism that prevents the development of cancer, including the death of transformed cells in the early stages of the disease, protective autophagy prevents apoptosis in tumor cells, promoting tumor metastasis and the development of resistance to radiation and chemotherapeutic agents, for example, the progression of endometrial cancer is associated with increased autophagy [95,98].

Lactic acid is an epigenetic regulator of gene activity, blocks HDAC, and enhances the transcription of genes responsible for components of the innate immune system, such as neutrophil gelatinase-associated lipocalin (NGAL). NGAL is present in neutrophils and many other tissues. Produced by epithelial cells in the presence of lactobacilli, NGAL suppresses bacterial siderophores, preventing the acquisition of iron by microorganisms, thereby inhibiting their growth, except for lactobacilli. D-lactic acid—a specific bacterial metabolite—is a more potent HDAC inhibitor compared to the L-isomer [96,99]. HDACs control the repression of non-expressed genes and also have a repressive effect on actively expressed genes. As a result, there is a weakening of transcription in active genes. Lactate, as an HDAC inhibitor, is able to preserve the transcription of active genes in order to avoid temporary glycogen depletion caused by increased glycolysis and maintains cell viability, which is also important for tumor cells. As experimental data show, inhibition of HDAC by lactic acid occurs at concentrations found in nature [100].

It has been shown that lactic acid produced by the bacteria *Lactobacillus*, *Lactococcus*, *Leuconostoc*, *Pediococcus*, *Streptococcus,* and *Peptostreptococcus* is involved in the development of oral squamous cell carcinoma by lowering the pH of the environment, enhancing gene transcription and DNA repair by inhibiting HDAC. It is likely that HDAC inhibition of actively expressed genes contributed to the survival of tumor cells. It is also noteworthy that the number of *Lactobacillus* has increased in accordance with the TNM stage increase in cancer patients after radiation and chemotherapy. Thus, by inducing selective gene transcription as an HDAC inhibitor, lactic acid can promote the survival of malignantly transformed cells and stimulate oncogenesis [101,102].

Secondary bile acids. Primary bile acids (BA)—cholic (CA) and chenodeoxycholic (CDCA) acids synthesized by liver cells—go through the enterohepatic circulation in the human body; one of the stages of which is the formation of secondary bile acids. Deoxycholic acid (DCA) and lithocholic acid (LCA) are formed, respectively, from CA and CDCA as a result of bacterial enzymatic dehydroxylation reactions in the large intestine [103]. The main producers of DCA and LCA are intestinal commensals of the *Clostridium* genus, which are part of cluster XVI (*Clostridium* cluster XVI): *C. scindens*, *C. hylemonae*, and cluster XI (*Clostridium* cluster XI): *C. sordelli*, *C. bifermentans*, *C. hiranonis* (*Peptacetobacter hiranonis*) [104,105].

The deconjugated primary bile salts cholate and chenodeoxycholate undergo reductive 7-dehydroxylation and are used by bacteria as electron acceptors, forming NAD+. Electron utilization by 7-dehydroxylating bacteria as a result of the conversion of primary FAs into secondary FAs provides this group of bacteria with a selective advantage in the process of colonization of the host’s intestine [106].

Secondary Bas—more hydrophobic and toxic than primary Bas—inhibits the growth of many intestinal bacteria by damaging their membranes, acting as detergents. Thus, the minimum inhibitory concentration of DCA against *Lactobacillus* and *Bifidobacterium* species is ten times lower than that of CA [107]. To reduce the toxicity of bile acids, intestinal microorganisms oxidize the hydroxyl groups of both primary and secondary BAs with the help of microbial HSDH (hydroxysteroid dehydrogenase) enzymes. However, some 7α-dehydroxylating bacteria have developed additional mechanisms that maintain antagonistic interactions with competing bacterial species by repeated reduction of the oxidized form of LCA (12-oxoLCA) [106,108].

In addition, the ability to produce antibacterial compounds helps 7α/β-dehydroxylating bacteria occupy their ecological niche in the large intestine. *C. scindens* has been shown to secrete tryptophan-derived antibiotics that inhibit the growth of *C. difficile*. The secretion of antibacterial compounds is enhanced by DCA and LCA, especially DCA, and these compounds themselves increase the toxicity of bile acids and the effectiveness of the suppression of intestinal bacteria [109]. In addition, secondary bile acids negatively affect spore germination, growth kinetics, and toxin activity of clinically significant strains of *C. difficile*, preventing the development of diseases associated with this bacterium [110]. Bile acids can also have an indirect effect on pathogens of intestinal infections by changing the composition of the microbiota. In experiments to prevent the colonization of chickens by the causative agent of campylobacteriosis *C. jejuni*, DCA was used, which had no inhibitory effect on *C. jejuni* in vitro. As a result, DCA-treated birds had a decreased amount of *Firmicutes*, an increased amount of *Bacteroidetes*, and decreased *C. jejuni* colonization [105].

Protecting the host from pathogens, DCA and LCA are toxic not only to the microbiota, but also to the host. Most LCA is esterified with sulfate in the human liver and excreted in the bile [111]. Due to the lack of a metabolic pathway to remove DCA, it accumulates in the bile acid pool. The amount of DCA in the pool depends on the rate of formation and absorption of DCA through the colon, the transit time, and the pH of the colon. With an increase in pH, the solubility of DCA increases, and therefore it is easily absorbed in the colon and enters the bloodstream. With a decrease in pH, an excess amount of deoxycholic acid can bind to dietary fiber and be excreted from the body [112].

The toxicity of secondary bile acids is also associated with carcinogenesis. They act as tumor promoters by modulating intracellular signaling and gene expression. DCA reduces p53 protein accumulation by stimulating proteasomal degradation. Being in a suppressed state, p53 and its response to DNA damaging agents such as ionizing radiation can manifest itself as the inability of cells to eliminate tumor transformation [113]. Interactions between DNA in Pirc rats with a germline mutation in the Apc—a tumor suppressor gene (TSG)—and the gut microbiota during carcinogenesis have been researched. The appearance of adenomas in the Pirc mucosa was associated with increased oxidative DNA damage, which was likely related to gut microbiota metabolism. Notably, the Pirc colon was enriched in 7-dehydroxylated bacteria *Clostridium* cluster XI [114]. In addition to oxidative damage to DNA by secondary BA associated with the formation of ROS and nitrogen, the impact of increasing concentrations of DCA on colonic epithelial cell cultures led to the formation of apoptosis resistance, and in experiments with animals, an increase in the expression of the anti-apoptotic protein Bcl-xL in the area adjacent to adenocarcinomas [115]. Secondary BAs are able to induce cancer stem cells (CSC) in colon epithelial cells. Given the key role of the Wnt/β-catenin signaling pathway in the regulation of colonic CSC proliferation, it has been shown that under the influence of DCA and LCA in HCoEpiC cells, the level of CSC markers (CD44 and CD166) has increased, as well as the expression of M3R, β-catenin, c-Myc, indicating cell transformation into CSCs by modulating M3R and Wnt/β-catenin signaling [116]. Microbial metabolites may be involved in the epigenetic regulation of cellular processes by influencing miRNA expression. Deoxycholic acid promotes the development of CRC by downregulating miR-199a-5p, which acts as an anti-oncogenic miRNA, inhibiting the proliferation, migration, and invasion of colorectal cancer cells in vitro and in vivo indirectly through CAC1 protein. This protein interacts with CDK2, activating the cell cycle, and the loss of CAC1 properties leads to its arrest in the G1/S phase [117,118].

## 6. Conclusions

In the course of co-evolution with macroorganisms, bacteria have developed effective mechanisms of interaction and adapted to their specific niches. The role of many commensals is determined by traditional ideas about their useful functions and maintaining homeostasis of the body’s biotopes, mainly through the synthesis of biologically active compounds. However, the survival of microorganisms and their successful colonization of various organs and systems of the host may be associated with the development of pathological effects, including the development and progression of tumors.

Microorganisms that synthesize cyclomodulins do little harm to the cells of the body in order to colonize it for a long time. Under unfavorable external conditions and a disturbed internal environment of the host, the effect of toxins on the eukaryotic cells of the body leads to DNA damage, disruption of the cell cycle, and mutational events that contribute to carcinogenesis.

Bacteria producing butyrate, lactic acid, and secondary bile acids limit exogenous microbial colonization either by the direct effect of these compounds on competing microorganisms or through host defense mechanisms or antimicrobial agents whose activity is associated with these metabolites. The same metabolites, in response to environmental factors, altered genetic background of the macroorganism, phenotypic, physiological, and biochemical diversity of its cells, can participate in carcinogenesis. The consequential imbalance between the state of the macroorganism and the factors of bacteria adaptation to it—cyclomodulins and some other microbial metabolites—can be the cause of the onset and development of malignant transformations in the host organism.

## Data Availability

Not applicable.
